# Genomic Resources and Tools for Gene Function Analysis in Potato

**DOI:** 10.1155/2008/216513

**Published:** 2008-12-18

**Authors:** Glenn J. Bryan, Ingo Hein

**Affiliations:** Genetics Programme, Scottish Crop Research Institute, Invergowrie, Dundee DD2 5DA, UK

## Abstract

Potato, a highly heterozygous tetraploid, is undergoing an exciting phase of genomics resource development. The potato research community has established extensive genomic resources, such as large expressed sequence tag (EST) data collections, microarrays and other expression profiling platforms, and large-insert genomic libraries. Moreover, potato will now benefit from a global potato physical mapping effort, which is serving as the underlying resource for a full potato genome sequencing project, now well underway. These tools and resources are having a major impact on potato breeding and genetics. The genome sequence will provide an invaluable comparative genomics resource for cross-referencing to the other Solanaceae, notably tomato, whose sequence is also being determined. Most importantly perhaps, a potato genome sequence will pave the way for the functional analysis of the large numbers of potato genes that await discovery. Potato, being easily transformable, is highly amenable to the investigation of gene function by biotechnological approaches. Recent advances in the development of Virus Induced Gene Silencing (VIGS) and related methods will facilitate rapid progress in the analysis of gene function in this important crop.

## 1. INTRODUCTION

Cultivated potato, the world's third most important human food crop, is a tetraploid outbreeder and suffers acutely from inbreeding depression. Genetic mapping is generally performed at the diploid level, using highly heterozygous clones as parents, and several diploid maps of potato have been generated [1], including one of the densest plant genetic maps [2]. Considerable progress has also been made in working at the tetraploid level [3, 4]. These efforts have led to the development of large numbers of molecular markers of all of the main types, which in some cases allow comparison of different potato maps or between potato and the closely related tomato. Genetic mapping has also led to knowledge of locations of many potato genes, notably those conferring resistance to many of the pests and pathogens that present a threat to potato [5] and genes influencing tuber traits [6]. Despite these advances, the lack of described mutational variation for potato is a disadvantage of its outbreeding mating habit, and renders genetic complementation problematic for the majority of genes. However, potato is relatively easy to transform, and so technologies such as overexpression and antisense technology are options for investigating gene function. Results of such experiments are not always so easy to interpret, and improved methods for functional analysis are critical to the future of potato breeding and genetics.

This article provides an overview of genomics resources currently available for potato, and the likely future developments in this area, paying particular emphasis to tools being developed for investigating gene function.

## 2. BASIC FACTS ABOUT THE POTATO GENOME

Cultivated potato has a chromosome number of 2*n* = 4*x* = 48, and a haploid genome size of ~850 Mb, roughly six times that of *Arabidopsis thaliana* and twice the size of the rice genome [7]. Although small chromosome size has been a limitation for cytogenetic analysis in potato, notable advances have been made using pachytene chromosomes and extended DNA “fibres” for fluorescence in situ hybridization (FISH) [8]. The potato genome is very similar in size to its close relative tomato, and genetic maps of the two species show high levels of macrocolinearity [9]. Information on how well the two genomes are conserved at the microsyntenic level should start to become available as outputs from the respective genome projects accumulate. The tomato genome mainly comprises low-copy-number sequences, which diverged rapidly in evolutionary time [10]. Schweizer et al. [11], who characterised the potato genome in terms of the amounts of different classes of repetitive DNA, suggest that the more highly repeated sequences comprise only 4–7% of the potato genome, suggesting that it was relatively devoid of highly repetitive DNA sequences, thus supporting the earlier tomato study. It is also known that the majority of tomato heterochromatin is found in centromeric regions with almost all of the euchromatic DNA located distally in long uninterrupted tracts, a structural feature likely to be true of potato [12]. Gene isolation and recent BAC-end sequencing efforts are providing the first detailed glimpses of the genome structure in potato. Using BAC-end sequence and full BAC sequence data, it has also been shown that potato (34%) contains considerably less repetitive DNA than tomato (46%), this difference being consistent with relative genome sizes of the two crops (850 versus 1000 Mb, resp.) [13].

## 3. STRUCTURAL GENOMICS RESOURCES FOR POTATO

### 3.1. EST resources

The generation of large expressed sequence tag (EST) collections is a primary route for large-scale gene discovery. There have been several efforts to generate EST resources for potato [14–16]. The potato gene index (http://compbio.dfci.harvard.edu/tgi/cgi-bin/tgi/gimain.pl?) contains almost 220000 ESTs, assembled into more than 30 000 “contigs” with over 26 000 singletons. These efforts, while not exhaustive, comprise a major genomics resource for potato researchers, perhaps comprising between 50–70% of the total potato gene “repertoire.” These ESTs will form an important source not only for the discovery of candidate genes and genetic markers, but also for the development of microarrays, until the whole genome sequence becomes available in potato. For instance, EST data from a number of different genotypes are also a rich source for the discovery of single nucleotide polymorphism (SNP) and simple sequence repeat (SSR) markers. For example, Tang et al. [17] demonstrate how large numbers of “eSNPs” can be mined from EST data using an SNP discovery pipeline (QualitySNP).

### 3.2. Large-insert genomic libraries and physical maps

Bacterial artificial chromosome (BAC) libraries have become the main vehicle for performing map-based gene cloning and physical mapping in potato. Several BAC libraries have been constructed from cultivated potato [18] and some of its wild relatives, for example, the diploids *Solanum bulbocastanum* [19]*, Solanum pinnatisectum* [20], and the Mexican hexaploid *Solanum demissum* [21]. These libraries represent a potentially useful resource for the study of comparative genome organisation and evolution in potato and the wider Solanaceae. A BAC library has been constructed from the male parent (RH89-039-16) of the cross used to make the ultra-high-density (UHD) genetic map of potato with 10 000 loci [2], and is being used for construction of a genome-wide potato physical map. Other significant developments arising from the use of these BAC libraries include the use of BAC clones and fluorescence in situ hybridization (FISH) to develop chromosome-specific cytogenetic DNA markers for chromosome identification in potato [22].

## 4. GENE ISOLATION IN POTATO

### 4.1. Map-based approach

Mapping efforts in potato have also led to the generation of knowledge concerning the genetic architecture of a number of characters, including pest and disease resistance, tuber quality traits, dormancy, tuber shape, and colour. Also, several potato genes have been isolated using a map-based approach [18, 23, 24], with most of these aimed at isolation of major genes for resistance to the more serious pests and pathogens of potato, the late blight pathogen *Phytophthora infestans* (Mont. de Bary), potato cyst nematodes (PCN), and potato virus X (PVX). These activities have necessitated the development of dense genetic maps around the target resistance loci, as well as concomitant generation of genomic resources, such as BAC libraries. These gene cloning efforts have afforded early glimpses into the structure of the potato genome, through the sequencing of a considerable number of large-insert clones. For instance, a study of *Gpa2/Rx1* resistance gene “cluster” provided important information concerning the evolution and structure of *R* gene loci and has shown beyond any doubt that resistances to different pests/pathogens can be coded by structurally similar genes from the same gene cluster.

The *R3* locus, which maps to a cluster of genes for resistance against *P. infestans* and other resistance genes on the short arm of chromosome XI, has shown to comprise two very tightly linked resistance genes (*R3a* and *R3b*) with distinct specificities against *P. infestans* [25]. The *R3* locus was found to be syntenic with the *I2* locus of tomato, and a comparative approach was used to isolate *R3a*, which is constitutively expressed along with some of its paralogous genes [26]. It is highly likely that the same approach will allow the future isolation of other *P. infestans* resistance genes on the same chromosome. Similarly, there are now determined efforts to isolate genes from late blight resistance “hotspots” on other potato chromosomes. A notable example is the recent work on potato chromosome IV, whereby several resistance genes against *P. infestans* map to the same locus [27–29].

These are but a few of several successful map-based gene isolation efforts, but these illustrate how comparative genomics, either between different potato genotypes or between different Solanaceous plant species, can be used as a tool for accelerating the normally laborious task of gene isolation, and they bode well for the future of Solanaceae genomic research. As knowledge of the genome structure of potato and tomato increases, the isolation of such genes should become more facile.

### 4.2. Candidate gene approach

A candidate gene approach has also been used for isolating plant genes that underlie specific traits [30]. In potato, cloning of the gene *Gro1-4*, which confers resistance to pathotype Ro1 of the cyst nematode *Globodera rostochiensis*, has been achieved using a joint candidate gene/mapping approach [31]. The gene was found to colocalise in a large segregating population with a marker derived from a “resistance-gene-like” sequence. The marker was used to isolate 15 members of a closely related gene family from genomic libraries. By taking into account all available information (inheritance patterns in resistant and susceptible germplasm, mapping data, DNA sequence information), it was possible to reduce the number of candidates to three genes, which were subsequently tested for complementation of a susceptible phenotype by stable transformation. The identified functional gene, a member of the TIR-NBS-LRR class, differs from susceptible members of the same family by 29 amino acid changes. This approach may be used in future for isolation of other resistance genes/QTLs conferring partial and durable resistance to the major potato pests and pathogens.

Another example of the use of candidate gene approach in potato is the isolation of *P* gene that encodes anthocyanin biosynthetic enzyme flavonoid 3′,5′-hydroxylase (f3′5′h), and is responsible for the production of blue/purple anthocyanin pigments in tissues like tubers, flowers, or stems [32]. In this study, a Petunia f3′5′h gene was used to screen a potato cDNA library prepared from purple-coloured flowers and stems. Six positively hybridizing cDNA clones were sequenced and all appeared to be derived from a single gene that shared 85% sequence identity at the amino acid level with Petunia f3′5′h. The potato gene cosegregated with purple tuber colour in a diploid population and was found to be expressed in tuber skin only in the presence of the anthocyanin regulatory locus I. One of the f3′5′h cDNA clone that was placed under the control of a doubled CaMV 35S promoter was also used for transformation of the red-skinned cultivar “Desiree.” Tuber and stem tissues that were coloured red in Desiree were purple in nine of 17 independently transformed lines, confirming the hypothesis that the transformed gene corresponded to the P locus.

In another study, DNA sequence variation was analysed at the invGE/GF locus (duplicate invertase genes *InvGE* and *InvGF*) on potato chromosome IX which colocalizes with a cold-sweetening QTL [33]. The study focused on 188 tetraploid potato cultivars, which were assessed for chip quality and tuber starch content. Two closely correlated invertase alleles, *invGE-f* and *invGF-d*, were associated with better chip quality in three breeding populations, and one allele (*invGF-b*) was associated with lower tuber starch content. The potato *invGE* gene was also found to be orthologous to the tomato invertase gene *Lin5*, causal for a fruit-sugar-yield QTL. These results suggested that natural variation for sugar yield in tomato fruits and that for sugar content in potato tubers are controlled by functional variants of orthologous invertase genes.

These few examples clearly demonstrate the potential of using the candidate gene approach in potato. It is also clear that the extensive knowledge of tuber biochemistry and the large number of potato gene sequences should enable its further application for tuber quality traits.

## 5. POTATO GENOME SEQUENCING

The ultra-high-density (UHD) genetic map of potato [2] forms the underlying framework for construction of a genome-wide physical map of the potato genome. Physical map construction is being carried out in two phases. First, approximately 73 000 clones from a BAC-library have been fingerprinted using a nonselective AFLP-based method. The fingerprint data has been used to assemble the RH BACs into roughly 7000 BAC contigs, with a similar number of “singletons” (i.e., single BAC clones). The second phase entails anchoring of the contigs and single BACs to the UHD map using a BAC pooling method, which should also reduce the number of contigs and increase the average contig size. Subsequent contiging will use a reduced stringency alignment approach which will reduce the number of contigs still further. The integrated genetic and physical map will be the main platform, which will be used for obtaining the DNA sequence of the potato genome. It is expected that approximately 1800 contigs will be anchored to the genetical map, and these scaffolds will be the starting point for genome sequencing. A BAC-end sequence resource, comprising more than 140 000 reads, has also been generated for the project [13]. The ongoing tomato and potato sequencing projects will have huge implications for those working in the Solanaceae, and will further sharpen the requirement for functional genomics tools.

## 6. ANALYSIS OF POTATO GENE EXPRESSION

A wide range of gene expression technologies have been used by potato researchers. Expression analysis is a discipline that is still very much in transition and it is likely to undergo significant development in the future, notably with recent developments in “next generation” sequencing (NGS) technologies, which have the potential to radically change the way gene discovery is performed.

### 6.1. cDNA-AFLP

The cDNA-AFLP technique has been used to study gene expression from stolon formation to sprouting in a range of different tissues during the potato tuber life cycle [34, 35]. Approximately 18 000 transcript-derived fragments (TDFs) were observed, and over 200 “process specific” TDFs belonging to different stages of potato tuber life cycle were isolated and sequenced. The sequence similarities of these TDFs to known genes give insights into the kinds of processes occurring during tuberisation, dormancy, and sprouting. This technique is extremely sensitive and can detect differences among gene family members indistinguishable by Northern blotting. A useful advance has been the realization that a large proportion of cDNA-AFLP fragments show genetic polymorphism in segregating populations and can be mapped as transcriptome-derived genetic markers [36]. Importantly, these markers show less centromeric clustering than AFLP markers derived directly from genomic DNA and appear to be targeted specifically to transcriptionally active regions of the genome. This method has been used to perform a large scale survey of genes differentially expressed during the tuber life cycle, and the isolation of some of their promoter regions [37]. Many genes expressed in the tuber life cycle are involved in defence, stress, storage, and signal transduction pathways. Twelve cis-acting elements were identified, and are known to be responsive to environmental stimuli known to play an important role during the tuber life cycle (light, sugars, hormones, etc.). More recently, a potato transcription map, based on cDNA-AFLP and containing approximately 700 TDFs, has been generated [38]. One of the disadvantages of cDNA-AFLP is that it does not provide gene sequence information and requires laborious isolation of gene fragments from polyacrylamide gels for sequence characterization.

### 6.2. SAGE

Serial analysis of gene expression (SAGE), which generates short cDNA sequence tags [39, 40] using a concatemerization-based method, has been used to examine global gene expression in potato tubers, generating 58 322 sequence tags (of length 19 nucleotides) of which 22 233 were unique [41]. Putative functions were assigned to almost 700 of those tags occurring at least ten times and roughly 70% matched each known potato EST sequence. This technology has the advantage over microarray technology in being an “open” technology, with the possibility of discovering “new” transcripts. Rapid amplification of complementary DNA ends (RACE) cloning was used to verify the reliability of SAGE tag annotation using EST sequences from more than one cultivar. Seventy two per cent of tags represented genes that participated in a known biological process, with the largest group (43%) consisting of transcripts active in physiological processes, about half of which were involved in metabolism. There were no transcripts found which were involved in photosynthesis. Of the 50 most abundant transcripts from the mature tuber, protease inhibitors were the dominant class, which is in good agreement with previous EST projects [14, 15].

The methodologies described briefly in this section are alternatives to the microarrays, which may ultimately be replaced by NGS methods. For example, Emrich et al. [42] recently demonstrated how such technologies can be used to extend significantly the EST resources for maize. The authors used a laser capture microdissection method to isolate rare transcripts from shoot apical meristems and then sequenced the corresponding cDNAs using 454 technology. This type of approach could be used in potato to identify transcripts not present in current EST databases or to extend the range of potato germplasm represented, currently limited to a few cultivars. All expression studies share the “problem” that they are only indicative of the function of particular genes or sets of genes in biological processes, and require functional analyses whereby the function of the candidate genes are compromised or exaggerated in some way (e.g., overexpression, silencing). This issue will be addressed in a subsequent section of this article.

## 7. MICROARRAYS: TOOLS FOR HIGH-THROUGHPUT GENE EXPRESSION ANALYSIS

### 7.1. cDNA microarrays

The available potato EST resources comprise an unknown but significant fraction of the gene complement of potato, and are derived from several genotypes, tissues, and environmental influences. A nonredundant set of 10 000 of these ESTs was used by the Institute for Genomic Research (TIGR) to develop a cDNA potato microarray that was made available to the research community at minimal cost. Moreover, the same organisation offered a transcription profiling service to allow the evaluation of these arrays by a wide range of users working on different Solanaceous plant species asking different biological questions. This allowed generation of massive microarray data that is publicly available (http://www.tigr.org/tdb/potato/profiling_service2.shtml#AProcedure). However, this platform had the disadvantage of containing a very small proportion of the potato gene repertoire. Moreover, as the “TIGR array” was based on spotted cDNAs, it was inherently difficult to achieve a high level of reproducibility. Rensink et al. [43] have used this platform to identify genes involved in abiotic stress responses, with more than 3 000 genes found to be significantly up- or downregulated in response to at least one of the stress conditions used (cold, heat, salt). In another detailed study, expression of 1315 genes during tuber development was examined, where transient changes in gene expression were found to be relatively uncommon and several new genes were found to be differentially expressed during tuber development [44]. These studies, while informative, highlight the dilemma faced by plant molecular biologists in prioritizing genes for further study from a large number of candidate genes in the absence of genetic information and mutations in target trait genes.

### 7.2. Oligonucleotide microarrays

Long oligonucleotide arrays that have been manufactured by various technology providers have also been found useful in potato since the use of short oligonucleotide arrays may lead to misinterpretations due to high degree of allelic heterozygosity in this crop. For this purpose, the potato oligo chip initiative (POCI) has selected the Agilent “44K feature platform” system, which was made available for use in 2006. This system is very flexible and allows for redesign of the array as more gene sequence information becomes available. Kloosterman et al. [45] described the design of this platform and demonstrated its utility by analyzing different stages of tuber initiation and growth.

## 8. FUNCTIONAL STUDIES IN POTATO

Potato geneticists and breeders have generated a great deal of information about the location of genes and QTLs coding for important potato traits, including pest and disease resistance and tuber traits. The volume of gene sequence information, notably from cDNA sequencing and the genome project, will increase rapidly in the coming years. Developments in genetics and structural genomics are beginning to be matched by concomitant development of functional genomics tools. Potato has a strong need for a high-density gene map or a genome sequence, to place gene sequences in their genetic/genomic context. Relatively high-throughput methods are also needed for testing and assessing gene function. The availability of mutant populations of potato will also be of tremendous value in this regard [46]. Potato cultivars are highly heterozygous and contain very high levels of “genetic load.” It has been estimated that there is one SNP approximately every 25 bp [47]. If individual alleles can be “isolated” in the homozygous condition, there is no telling what information they would yield about potato biology. The nonavailability of mutants may largely be overcome by recourse to use of diploid self-compatible potato clones for the development of mutant populations or by mining of variant alleles in heterozygous germplasm. Functional studies currently rely on the use of transformation-based techniques or use of viral vector-mediated gene delivery systems for the establishment of information regarding gene function. There have been some recent tantalising developments in functional genetics/genomics tools and resources for potato. Of course gene expression profiling or microarray studies have a role to play in the identification of a pool of candidate genes potentially involved in any given biological process. These methods, in combination with other functional genomics tools such as RNA interference (RNAi), virus-induced gene silencing (VIGS), and activation tagged lines, have the potential to facilitate the identification of the role of thousands of potato genes over the next several years. Furthermore, combining structural genetics approaches (such as QTL and candidate gene mapping) with functional genomics information (such as microarray-derived gene expression data for candidate genes) has great potential for the dissection of many complex, polygenic potato traits.

### 8.1. Virus-induced gene silencing (VIGS)

Virus-induced gene silencing (VIGS) is a powerful tool for plant functional genomics. VIGS exploits an RNA-mediated antiviral defense mechanism in plants. This phenomenon has been exploited for gene silencing through the use of virus vectors carrying host target genes that are directed against the corresponding plant mRNAs [48]. VIGS is increasingly used to generate transient loss-of-function assays, and is a powerful reverse-genetics tool in functional genomic programs as an alternative to stable transformation. In potato, two viral vectors, potato virus X (PVX) and tobacco rattle virus (TRV), have been successfully utilized for VIGS [49, 50]. Faivre-Rampant et al. [49] have shown that a binary PVX-based vector, pGR106, [51, 52] is effective in triggering VIGS of phytoene desaturase (PDS) in both diploid and cultivated tetraploid *Solanum* species. In this study, silencing was maintained throughout the foliar tissues and tubers and could also be triggered and sustained in in vitro micropropagated tetraploid potato for several cycles and on in vitro generated microtubers. Similarly, PDS silencing with TRV has been observed in cultivated potato, as well as the diploid wild species *S. bulbocastanum* and *S. okadae*, and the distantly related hexaploid *S. nigrum* [50]. In the same study, silencing of known resistance genes (e.g., *R1*, *Rx*, and *RB*) in normally resistant plants yielded a compatible interaction in detached leaf tests. A modification of the leaf inoculation used for both PVX- and TRV-based silencing was demonstrated for TRV in a so-called “agrodrench” method, in which soil adjacent to the plant root is drenched with an Agrobacterium suspension carrying the TRV-derived VIGS vectors [53]. TRV-based silencing of genes such as PDS, a 20S proteasome subunit (PB7) or Mg-protoporphyrin chelatase (Chl H) by agrodrench has been shown to be efficient for different members of the Solanaceae including *Nicotiana benthamiana*, tomato, pepper, tobacco, potato, and petunia.

**Figure 1:**
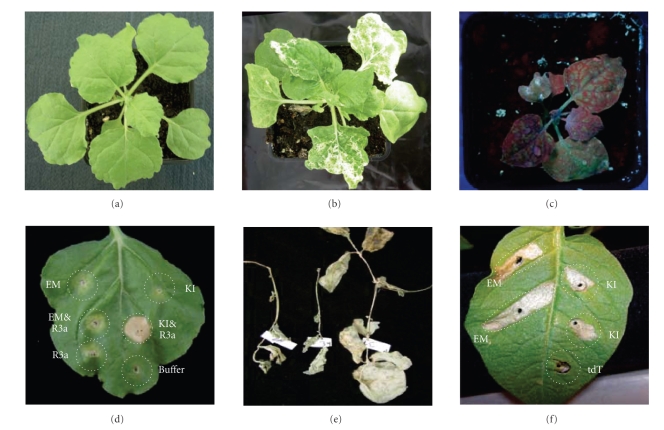
*N. benthamiana* plants, noninoculated (a), inoculated with pGR106::PDS to silence endogenous phytoene desaturase resulting in photo bleaching of leaves (b) and overexpressing GFP via a pGR106::GFP construct—viewed under UV light to show expression of GFP (c). Overexpression and coinfiltration of virulent Avr3a KI and avirulent Avr3a EM with the potato *R* gene R3a are shown in (d). Naturally occurring PVX resistance in *S. papita* (e) and the recognition of virulent Avr3a KI and avirulent Avr3a EM alleles but not from tdT, used as a control, in *S. chacoense* (f).

*N. benthamiana* provides a particularly suitable model system for Solanaceae species, including potato, as it is highly amenable to manipulations such as VIGS and virus- or Agrobacterium-mediated overexpression of candidate genes (Figure 1) [54]. Indeed, many silencing studies have been conducted in *N.* *benthamiana* to demonstrate involvement of candidate genes involved in the plant disease resistance (including the hypersensitive response; HR), abiotic stress, cellular signaling, and secondary metabolite biosynthesis [55]. Recently, for example, Gilroy et al. [56], using a combination of VIGS and biochemical approaches, demonstrated that the cysteine protease cathepsin B is required for the HR. Silencing of cathepsin B in *N. benthamiana* prevented programmed cell death (PCD) and compromised disease resistance induced by *Erwinia amylovora* and *Pseudomonas syringae* pv. tomato (Pst) DC3000, two distinct nonhost bacterial pathogens. It also suppressed the HR triggered by transient coexpression of potato *R3a* and *Phytophthora infestans* *Avr3a* genes but did not compromise the HR triggered by recognition of *Cladosporium* *fulvum* AVR4 by tomato Cf-4. The ease of silencing in *N. benthamiana* makes it suitable for large scale VIGS experiments. A study of 192 cDNA-AFLP fragments, expressed during the HR following recognition of *Avr4* from *C. fulvum* by tomato *Cf-4*, was conducted in *N. benthamiana* and identified 15 *Avr4*-responsive tomato (ART) fragments that, when silenced, resulted in a compromised HR induced by both *Avr4* in Cf-4 transgenic plants and the *Inf1* gene from *P. infestans* [57]. In addition, silencing of HSP90, a nuclear GTPase, an L19 ribosomal protein, and a nucleotide binding-leucine rich repeat (NB-LRR)-type protein suppressed the HR [57]. Interestingly, silencing of the NB-LRR-type protein NRC1 not only affected the Cf-4/Avr4-induced HR and compromised Cf-4-mediated resistance to *C. fulvum*, but also revealed that this protein is required for the HR induced by the *R* proteins Cf-9, LeEix, Pto, Rx, and Mi [58].

A recently developed TRV RNA2 vector, which utilizes ligation-independent cloning (LIC), has been employed to assess the function of 400 tomato ESTs in *N. benthamiana* [59]. The function of SlMADS1 and its *N. benthamiana* homologous sequences, NbMADS4-1 and -2, was shown during flowering and demonstrated that NbMADS4-1 and NbMADS4-2 act nonredundantly in floral development. Silencing of either gene resulted in loss of organ identity. These studies show the potential for use of *N. benthamiana* as a “proxy” species for high-throughput gene function analysis for potato and other Solanaceae.

### 8.2. Virus and Agrobacterium tumefaciens-based overexpression

In addition to their role in VIGS, virus vectors can be used for overexpressing genes in plants. The Agrobacterium PVX-based binary vector pGR106, an efficient silencing vector for Solanum species, can also be used for overexpressing genes, as shown for GFP in Figure 1 [51, 52]. The search for novel sources of plant resistance, driven by knowledge of pathogen “effectors” with avirulent activities, rather than more traditional plant disease resistance breeding, has been coined “effectoromics” [60]. For example, overexpression of *P. infestans* effectors in potato represents an opportunity to seek vital and invariant components of the *P. infestans* pathogenicity apparatus that can be targeted for sustainable potato protection. Information emerging from effectoromic studies will be useful to identify the cognate host *R* genes as sources of durable disease resistance and to develop novel control strategies that are intrinsically difficult for the pathogen to overcome. The discovery of a conserved motif, RxLR, within many avirulence genes [61, 62] that is required for translocation of the effectors from pathogen haustoria into the plant cell [63] has had a tremendous impact on the prediction of pathogen effectors. Overexpression via pGR106 in *N. benthamiana* of 63 predicted *P.* *infestans* extracellular proteins (Pex) led to the discovery of two novel necrosis-inducing cDNAs, encoding extracellular proteins belonging to a large and complex protein family in *Phytophthora* [64]. Similarly, the recognition of the *P. infestans* effector *Avr3a* by the potato *R* gene *R3a* [26] could be demonstrated in *N. benthamiana* [61]. Coinfiltration of *N. benthamiana* leaves with an *A. tumefaciens* strain carrying a construct expressing *R3a* and a strain carrying a construct expressing the truncated avirulent *Avr3a* (*Avr3a* KI) sequence via PVX resulted in a confluent cell death response, not observed when overexpressing the truncated virulent *Avr3a* (*Avr3a* EM) sequence (Figure 1). Using the *P. infestans* elicitins INF1, INF2A, and INF2B, the same PVX system has been adapted and optimized to screen Solanum plants for response to pathogen elicitors [65]. Of 31 potato species tested, 11 clones of *Solanum huancabambense* and *Solanum microdontum* responded with HR-like symptoms, which were also observed following infiltration with purified recombinant INF1, INF2A, and INF2B.

Two similar studies have been reported that utilize the two *Avr3a* alleles described above to identify potentially novel resistance mechanisms within wild potato accessions [66, 67]. One study [66] utilized PVX to express the different *Avr3a* alleles in wild Solanum species, whereas the other [67] utilized Agrobacterium-only-based expression of the *Avr3a* alleles to circumvent the relative high level of resistance against PVX within the wild species tested (Figure 1). These studies identified similar sets of species that recognize both the EM and KI forms of AVR3a (unpublished data).

## 9. WHERE NEXT FOR POTATO?

Potato has entered an exciting new era, whereby the development of extensive genetic and genomic resources have opened up many new possibilities for studying important potato traits relevant to potato agronomy. Concomitant development of similar resources for other Solanaceous species, notably tomato, and a growing cohesiveness of the Solanaceae research community, as demonstrated by the “SOL vision” (http://www.sgn.cornell.edu/solanaceae-project/) bode well for future genomic research of potato and its close relatives. Development of biotechnological tools for assaying potato gene function is likely to progress rapidly in the coming years.
